# 

**Published:** 1911-01-21

**Authors:** 


### CONTENTS.

| | PAGE |
|---|---|
| **The Responsibility at Law of the Parent** ... | 487 |
| **The Thymus Treatment of Inoperable Cancer** ... | 488 |
| **Annotations**— | |
| The International Committee for Graduate Instruction in Medicine—Mr. Ryall's Exoneration—High Tea—Consultant and G.P. ... | 489 |
| **Medical Opinion and Movement** ... | 490 |
| **Hospital Clinics**— | |
| Drug Eruptions: their Nature and Varieties. By J. H. Stowers, M.D. ... | 493 |
| **Medicine**— | |
| Gas in the Stomach and its Treatment ... | 495 |
| **Tropical Medicine**— | |
| Bilharziosis of the Appendix ... | 498 |
| Dermatobia Noxialis ... | 498 |
| **The General Practitioner's Column**— | |
| Treatment of Fistula in Ano ... | 499 |
| **International Committee for Post-Graduate Medical Education** ... | 499 |
| **"The Hospital" Medical Book Supplement—No. XXXVIII.** ... | 501 |
| **News and Events of the Week** ... | 505 |
| **The Week in the Courts** ... | 505 |
| **Parliament and Publications** ... | 506 |
| **Editor's Letter-Box**— | |
| A Year's Progress in Animal Protection ... | 507 |
| **Medical Appointments, &c.** ... | 507 |
| **Australian Notes** ... | 508 |
| **The Week's Work**— | |
| Co-operation in Contracts—Geographical Limitations—The Construction of Hospitals in the Tropics—The Hospital Plan in Warm Climates—How to Help the Hospitals—This Week's Drug Market ... | 509 |
| **Special Institutional Articles**— | |
| The Proper Relation of the Superintendent to the Trustees of a Hospital... | 511 |
| Sanatorium Treatment for the Poor ... | 512 |
| Hospitals and the Law in 1910 ... | 513 |
| **Gifts and Bequests** ... | 514 |
| **Hospital Architecture and Construction**— | |
| The Ward Floor ... | 515 |
| **Institutional Notes and News** ... | 515 |
| **The Hospital World** ... | 517 |
| **Jottings** ... | 517 |
| **New Appliances and Things Medical** ... | 518 |
| **Obituary** ... | 518 |
| **The Week's Novelties** ... | 518 |

